# 2-Methyl-2-(2-pyrid­yl)hexa­hydro­pyrimidine

**DOI:** 10.1107/S1600536809025963

**Published:** 2009-07-15

**Authors:** Saud Al-Resayes

**Affiliations:** a Department of Chemistry, King Saud University, PO Box 2455, Riyadh 11451, Saudi Arabia

## Abstract

In the aminal-type title compound, C_10_H_15_N_3_, the six-membered hexa­hydro­pyrimidine ring adopts a chair conformation and the N atoms are pyramidally coordinated. One of the two amido –NH units engages in inter­molecular hydrogen bonding with the pyridyl N atom, generating a helical chain running along the *b* axis of the ortho­rhom­bic unit cell.

## Related literature

The title compound is used in Fe(II) spin-crossover materials; see: Bréfuel *et al.* (2007 ▶).
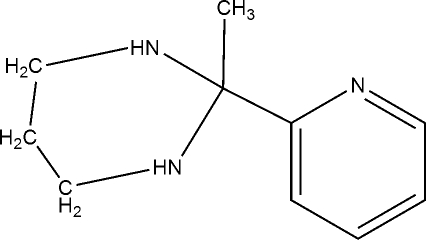


## Experimental

### 

#### Crystal data

C_10_H_15_N_3_
*M* *_r_* = 177.25
Orthorhombic, 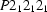

*a* = 8.4070 (17) Å
*b* = 10.371 (2) Å
*c* = 11.363 (2) Å
*V* = 990.7 (3) Å^3^
*Z* = 4
Mo *K*α radiation
μ = 0.07 mm^−1^
*T* = 294 K
0.35 × 0.15 × 0.15 mm

#### Data collection

Rigaku R-AXIS RAPID diffractometer
Absorption correction: multi-scan (*CrystalClear*; Rigaku/MSC, 2007 ▶) *T*_min_ = 0.97, *T*_max_ = 0.99
8017 measured reflections
1324 independent reflections
1206 reflections with *I* > 2σ(*I*)
*R*_int_ = 0.022

#### Refinement

*R*[*F* ^2^ > 2σ(*F* ^2^)] = 0.044
*wR*(*F* ^2^) = 0.115
*S* = 1.01
1324 reflections
128 parameters
H atoms treated by a mixture of independent and constrained refinement
Δρ_max_ = 0.20 e Å^−3^
Δρ_min_ = −0.34 e Å^−3^

### 

Data collection: *CrystalClear* (Rigaku/MSC, 2007 ▶); cell refinement: *CrystalClear*; data reduction: *CrystalClear*; program(s) used to solve structure: *SHELXS97* (Sheldrick, 2008 ▶); program(s) used to refine structure: *SHELXL97* (Sheldrick, 2008 ▶); molecular graphics: *DIAMOND* (Brandenburg, 2006 ▶) and *PLUTO* (Motherwell *et al.*, 1999 ▶); software used to prepare material for publication: *publCIF* (Westrip, 2009 ▶).

## Supplementary Material

Crystal structure: contains datablocks I, global. DOI: 10.1107/S1600536809025963/ng2608sup1.cif

Structure factors: contains datablocks I. DOI: 10.1107/S1600536809025963/ng2608Isup2.hkl

Additional supplementary materials:  crystallographic information; 3D view; checkCIF report

## Figures and Tables

**Table 1:** Hydrogen-bond geometry (Å, °)

*D*—H⋯*A*	*D*—H	H⋯*A*	*D*⋯*A*	*D*—H⋯*A*
N2—H2*A*⋯N1^i^	0.89 (3)	2.31 (3)	3.188 (2)	168 (2)

Symmetry code: (i) 
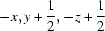
.

**Fig. 1.**
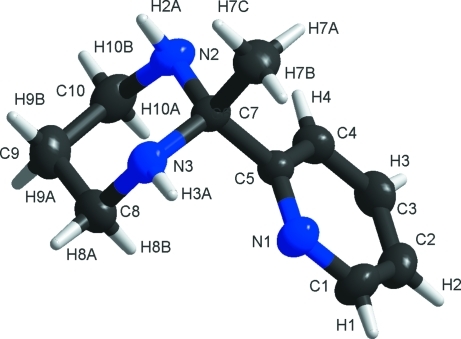
Perspective drawings of the title compound showing the atom-numbering scheme. The atomic displacement ellipsoids are shown at the 50% probability level. Hydrogen atoms are drawn as spheres of arbitrary radius.

**Fig. 2.**
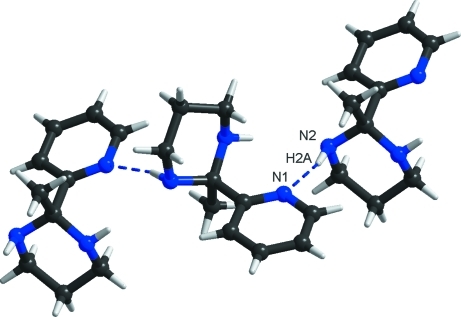
N— H···N intermolecular hydrogen bonding pattern of the title compound with hydrogen bonding shown as broken lines. Symmetry code: -x, y + 1/2, -z + 1/2.

## supplementary crystallographic information

### Comment

The title compound was known as precursor for syntheses of umsymmetrical tetradentate Schiff base ligands that form bistable Fe(II) spin crossover materials (Bréfuel *et al. *2007).

The molecular packing of the title compound is supported by N— H···N intermolecular hydrogen bondings at H···A distance of 2.31 (3) and D— H···A angle of 168 (2)° and calculated with Pluto (Motherwell *et al.*, 1999), see Figure 2.

### Experimental

1,3-Propane diamine (1 g, 0.013 mmol) was mixed with 1-(2-pyridinyl)-1-ethanone (1.635 g, 0.013 mmol) in 30 ml e thanol. The mixture was stirred under reflux for 5 h. The solution was concentrated under reduced pressure and the product was precipitated by addition of 30 ml of cool distilled water. Product was filtered off and washed three times with 15 ml of distilled water then dried under vacuum. Crude product was recrystallized from ethanol and allowed to stand at room temperature. Crystals were collected after 2 weeks.

### Refinement

Hydrogen atoms were refined isotropically and were constrained to the ideal geometry using an appropriate riding model with *U*_iso_(H) fixed at 1.2 times *U*_eq_ of the pivot atom. The —NH hydrogen atoms was located from difference Fourier map and refined isotropically without constraints. 949 Friedel-pair reflections were merged for a weak anomalous scatterer structure.

### Crystal data

**Table:**

C_10_H_15_N_3_	*F*(000) = 384
*M**_r_* = 177.25	*D*_x_ = 1.188 Mg m^−^^3^
Orthorhombic, *P*2_1_2_1_2_1_	Mo *K*α radiation, λ = 0.71073 Å
Hall symbol: P 2ac 2ab	Cell parameters from 7168 reflections
*a* = 8.4070 (17) Å	θ = 3.0–27.5°
*b* = 10.371 (2) Å	µ = 0.07 mm^−^^1^
*c* = 11.363 (2) Å	*T* = 294 K
*V* = 990.7 (3) Å^3^	Plate, yellow
*Z* = 4	0.35 × 0.15 × 0.15 mm

### Data collection

**Table:**

Rigaku R-AXIS RAPID diffractometer	1324 independent reflections
Radiation source: fine-focus sealed tube	1206 reflections with *I* > 2σ(*I*)
graphite	*R*_int_ = 0.022
ω scans	θ_max_ = 27.5°, θ_min_ = 3.0°
Absorption correction: multi-scan (*CrystalClear*; Rigaku/MSC, 2007)	*h* = −10→10
*T*_min_ = 0.97, *T*_max_ = 0.99	*k* = −13→12
8017 measured reflections	*l* = −13→14

### Refinement

**Table:**

Refinement on *F*^2^	Primary atom site location: structure-invariant direct methods
Least-squares matrix: full	Secondary atom site location: difference Fourier map
*R*[*F*^2^ > 2σ(*F*^2^)] = 0.044	Hydrogen site location: inferred from neighbouring sites
*wR*(*F*^2^) = 0.115	H atoms treated by a mixture of independent and constrained refinement
*S* = 1.01	*w* = 1/[σ^2^(*F*_o_^2^) + (0.0907*P*)^2^ + 0.0352*P*] where *P* = (*F*_o_^2^ + 2*F*_c_^2^)/3
1324 reflections	(Δ/σ)_max_ < 0.001
128 parameters	Δρ_max_ = 0.20 e Å^−^^3^
0 restraints	Δρ_min_ = −0.34 e Å^−^^3^

### Special details

**Table:**

Geometry. All e.s.d.'s (except the e.s.d. in the dihedral angle between two l.s. planes) are estimated using the full covariance matrix. The cell e.s.d.'s are taken into account individually in the estimation of e.s.d.'s in distances, angles and torsion angles; correlations between e.s.d.'s in cell parameters are only used when they are defined by crystal symmetry. An approximate (isotropic) treatment of cell e.s.d.'s is used for estimating e.s.d.'s involving l.s. planes.
Refinement. Refinement of *F*^2^ against ALL reflections. The weighted *R*-factor *wR* and goodness of fit *S* are based on *F*^2^, conventional *R*-factors *R* are based on *F*, with *F* set to zero for negative *F*^2^. The threshold expression of *F*^2^ > σ(*F*^2^) is used only for calculating *R*-factors(gt) *etc*. and is not relevant to the choice of reflections for refinement. *R*-factors based on *F*^2^ are statistically about twice as large as those based on *F*, and *R*- factors based on ALL data will be even larger.

### Fractional atomic coordinates and isotropic or equivalent isotropic displacement parameters (Å^2^)

**Table:**

	*x*	*y*	*z*	*U*_iso_*/*U*_eq_	
N1	0.20810 (17)	0.34068 (14)	0.21846 (15)	0.0473 (4)	
C1	0.3442 (2)	0.27459 (18)	0.2066 (2)	0.0562 (5)	
H1	0.3416	0.1859	0.2183	0.067*	
N2	0.05820 (18)	0.66511 (13)	0.16234 (14)	0.0475 (3)	
H2A	−0.026 (3)	0.711 (2)	0.187 (2)	0.063 (6)*	
C2	0.4872 (2)	0.3304 (2)	0.17791 (18)	0.0553 (5)	
H2	0.5789	0.2809	0.1707	0.066*	
N3	−0.08570 (15)	0.46514 (15)	0.18351 (14)	0.0436 (3)	
H3A	−0.087 (3)	0.391 (2)	0.221 (2)	0.063 (6)*	
C3	0.49114 (19)	0.4615 (2)	0.16021 (17)	0.0518 (4)	
H3	0.5861	0.5025	0.1410	0.062*	
C4	0.35197 (19)	0.53176 (17)	0.17129 (16)	0.0445 (4)	
H4	0.3523	0.6205	0.1599	0.053*	
C5	0.21182 (17)	0.46810 (14)	0.19969 (13)	0.0364 (3)	
C6	0.05402 (18)	0.53948 (14)	0.22108 (14)	0.0385 (3)	
C7	0.0394 (3)	0.5618 (2)	0.35388 (16)	0.0574 (5)	
H7A	0.1299	0.6097	0.3812	0.086*	
H7B	0.0352	0.4802	0.3936	0.086*	
H7C	−0.0560	0.6095	0.3701	0.086*	
C8	−0.0901 (2)	0.44394 (19)	0.05613 (18)	0.0536 (4)	
H8A	−0.1817	0.3918	0.0356	0.064*	
H8B	0.0051	0.3987	0.0311	0.064*	
C9	−0.1001 (3)	0.5740 (2)	−0.00463 (18)	0.0630 (5)	
H9A	−0.0978	0.5624	−0.0893	0.076*	
H9B	−0.1990	0.6164	0.0161	0.076*	
C10	0.0403 (2)	0.6562 (2)	0.03398 (18)	0.0574 (5)	
H10A	0.1370	0.6202	0.0009	0.069*	
H10B	0.0274	0.7424	0.0022	0.069*	

### Atomic displacement parameters (Å^2^)

**Table:**

	*U*^11^	*U*^22^	*U*^33^	*U*^12^	*U*^13^	*U*^23^
N1	0.0406 (7)	0.0382 (6)	0.0631 (9)	0.0007 (6)	0.0038 (7)	0.0057 (6)
C1	0.0532 (10)	0.0419 (8)	0.0737 (12)	0.0105 (8)	0.0012 (9)	0.0040 (9)
N2	0.0480 (7)	0.0342 (6)	0.0602 (8)	0.0046 (6)	0.0084 (7)	0.0023 (6)
C2	0.0406 (8)	0.0658 (11)	0.0594 (10)	0.0145 (8)	0.0010 (7)	−0.0043 (9)
N3	0.0321 (6)	0.0439 (7)	0.0549 (8)	−0.0012 (5)	0.0053 (5)	0.0062 (7)
C3	0.0340 (7)	0.0649 (10)	0.0564 (9)	−0.0048 (7)	0.0033 (7)	−0.0035 (9)
C4	0.0388 (7)	0.0425 (7)	0.0522 (9)	−0.0059 (6)	0.0040 (6)	−0.0022 (7)
C5	0.0340 (7)	0.0353 (7)	0.0400 (7)	−0.0006 (6)	0.0011 (6)	−0.0017 (6)
C6	0.0371 (7)	0.0359 (7)	0.0427 (7)	0.0006 (6)	0.0067 (6)	0.0007 (6)
C7	0.0614 (11)	0.0643 (11)	0.0466 (9)	0.0089 (9)	0.0088 (8)	−0.0057 (8)
C8	0.0415 (8)	0.0590 (10)	0.0603 (10)	−0.0022 (8)	−0.0019 (7)	−0.0084 (8)
C9	0.0568 (11)	0.0812 (13)	0.0509 (10)	0.0073 (10)	−0.0036 (8)	0.0088 (10)
C10	0.0591 (10)	0.0551 (9)	0.0579 (10)	0.0062 (9)	0.0097 (9)	0.0181 (9)

### Geometric parameters (Å, °)

**Table:**

N1—C1	1.340 (2)	C4—H4	0.9300
N1—C5	1.339 (2)	C5—C6	1.539 (2)
C1—C2	1.374 (3)	C6—C7	1.532 (2)
C1—H1	0.9300	C7—H7A	0.9600
N2—C10	1.469 (3)	C7—H7B	0.9600
N2—C6	1.464 (2)	C7—H7C	0.9600
N2—H2A	0.89 (3)	C8—C9	1.517 (3)
C2—C3	1.375 (3)	C8—H8A	0.9700
C2—H2	0.9300	C8—H8B	0.9700
N3—C6	1.468 (2)	C9—C10	1.521 (3)
N3—C8	1.464 (3)	C9—H9A	0.9700
N3—H3A	0.88 (3)	C9—H9B	0.9700
C3—C4	1.384 (2)	C10—H10A	0.9700
C3—H3	0.9300	C10—H10B	0.9700
C4—C5	1.389 (2)		
			
C1—N1—C5	117.95 (14)	N2—C6—C5	109.60 (12)
N1—C1—C2	123.75 (16)	C7—C6—C5	107.30 (14)
N1—C1—H1	118.1	C6—C7—H7A	109.5
C2—C1—H1	118.1	C6—C7—H7B	109.5
C10—N2—C6	113.21 (14)	H7A—C7—H7B	109.5
C10—N2—H2A	105.3 (17)	C6—C7—H7C	109.5
C6—N2—H2A	108.1 (16)	H7A—C7—H7C	109.5
C3—C2—C1	118.18 (16)	H7B—C7—H7C	109.5
C3—C2—H2	120.9	N3—C8—C9	108.52 (16)
C1—C2—H2	120.9	N3—C8—H8A	110.0
C6—N3—C8	112.72 (13)	C9—C8—H8A	110.0
C6—N3—H3A	109.1 (16)	N3—C8—H8B	110.0
C8—N3—H3A	110.1 (16)	C9—C8—H8B	110.0
C2—C3—C4	119.15 (16)	H8A—C8—H8B	108.4
C2—C3—H3	120.4	C10—C9—C8	108.91 (16)
C4—C3—H3	120.4	C10—C9—H9A	109.9
C5—C4—C3	119.22 (16)	C8—C9—H9A	109.9
C5—C4—H4	120.4	C10—C9—H9B	109.9
C3—C4—H4	120.4	C8—C9—H9B	109.9
N1—C5—C4	121.74 (15)	H9A—C9—H9B	108.3
N1—C5—C6	115.44 (13)	N2—C10—C9	113.65 (16)
C4—C5—C6	122.65 (13)	N2—C10—H10A	108.8
N3—C6—N2	110.72 (13)	C9—C10—H10A	108.8
N3—C6—C7	107.56 (13)	N2—C10—H10B	108.8
N2—C6—C7	108.46 (14)	C9—C10—H10B	108.8
N3—C6—C5	113.03 (12)	H10A—C10—H10B	107.7

### Hydrogen-bond geometry (Å, °)

**Table:**

*D*—H···*A*	*D*—H	H···*A*	*D*···*A*	*D*—H···*A*
N2—H2A···N1^i^	0.89 (3)	2.31 (3)	3.188 (2)	168 (2)

Symmetry codes: (i) −*x*, *y*+1/2, −*z*+1/2.
